# Understanding Progestins: From Basics to Clinical Applicability

**DOI:** 10.3390/jcm12103388

**Published:** 2023-05-10

**Authors:** Manuel García-Sáenz, Raúl Ibarra-Salce, Francisco Javier Pozos-Varela, Tania Sofia Mena-Ureta, Susana Flores-Villagómez, Mario Santana-Mata, Ramón G. De Los Santos-Aguilar, Daniel Uribe-Cortés, Aldo Ferreira-Hermosillo

**Affiliations:** 1 Servicio de Endocrinología, Hospital de Especialidades del Centro Médico Nacional Siglo XXI, Instituto Mexicano del Seguro Social, Mexico City 06720, Mexico; manuel.gsm@hotmail.com; 2 Departamento de Endocrinología, Facultad de Medicina, Universidad Autónoma de Coahuila, Saltillo 25204, Mexico; dr.raulsalce@gmail.com; 3 Hospital Star Médica, Aguascalientes 20029, Mexico; paco.pozos.v@gmail.com (F.J.P.-V.); tania.menaureta@gmail.com (T.S.M.-U.); 4 Centro Médico Campestre, Leon 37180, Mexico; draspfv@gmail.com; 5 Departamento de Medicina Interna, Hospital General de Zona N. 2, Saltillo 25296, Mexico; santana.endocrinologia.md@gmail.com; 6 Departamento de Biología de la Reproducción Dr. Carlos Gual Castro, Instituto Nacional de Ciencias Médicas y Nutrición Salvador Zubirán, Mexico City 14080, Mexico; ramon.delossantosa@incmnsz.mx; 7 Hospital Ángeles Clinica Londres Torre Frontera, Mexico City 06700, Mexico; ponce484@hotmail.com; 8 Unidad de Investigación Médica en Enfermedades Endocrinas, Centro Médico Nacional Siglo XXI, Instituto Mexicano del Seguro Social, Mexico City 06720, Mexico

**Keywords:** progestins, progesterone congeners, progestogens, endometriosis, contraceptive agents, hormone replacement therapy, luteal phase

## Abstract

Progestin is a term used to describe a synthetic progestogen. The activity and potency of synthetic progestins are mostly evaluated via parameters associated with their endometrial effects, which are related to their interactions with progesterone, estrogen, androgen, glucocorticoid, and mineralocorticoid receptors. The chemical structure of progestins is the key to understanding their interactions with these receptors and predicting the other effects associated with these drugs. Due to their endometrial effect, progestins are used for different gynecological conditions, such as endometriosis, contraception, hormonal replacement therapy, and artificial reproduction techniques. This review is focused on improving our knowledge of progestins (from their history and biochemical effects related to their chemical structures to clinical applications in gynecological conditions) in order to improve clinical practice.

## 1. Introduction

**Figure 1:**
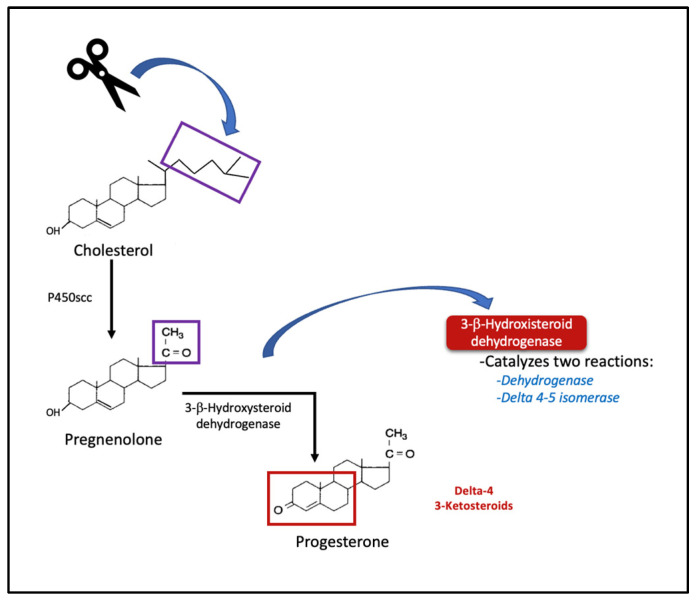
Synthesis of progesterone from cholesterol. Initial steps of steroidogenesis and delta-4 products. p450scc = cholesterol side chain cleavage enzyme.

Progesterone (P4) is a steroid hormone containing 21 carbons that is synthesized primarily by glands in the ovaries, adrenals, and testicles and plays a fundamental role in coordinating the reproductive physiology of females (ovulation, endometrium transformation, and pregnancy maintenance) [1,2]. It is synthesized from cholesterol, which is converted to the P4 precursor, pregnenolone, by the mitochondrial cholesterol side-chain cleavage enzyme (cytochrome P450scc), and then pregnenolone is converted to P4 in a reaction catalyzed by 3β-hydroxysteroid dehydrogenase (3-β-HSD) (see Figure 1) [3].

The activity of P4 is based on its interaction with P4 receptors (PR), a nuclear transcription factor, that acts via specific P4 response elements within the promoter area of its target genes to regulate transcription [4].

Progestagen is a term used to describe steroid hormones that have progestational effects, such as P4, and the term progestin refers only to synthetic progestational agents [5] that are developed to mimic the biological action of the endogenous P4. These agents have many therapeutic applications in female reproductive medicine and are used because they have better bioavailability and half-lives than P4. At present, various other properties of these agents have been described, which allow for their use in different therapeutic applications that include contraception, hormonal replacement therapy (HRT), and the treatment of gynecological disorders such as endometriosis and polycystic ovary syndrome [6].

## 2. Methods

We carried out a literature search up to January 2023 for publications related to P4 and progestins, which included general information on the function of P4, historical background on the discovery of P4 and the development of progestins, the classification of progestins, and their clinical applications in diseases related to reproductive endocrinology (endometriosis, contraception, menopausal therapy, and assisted reproductive techniques). The search was conducted in PubMed and only included publications with texts or abstracts available in English.

## 3. Progestin History

The story of the discovery and characterization of P4 is one of the typical historical accounts that exemplifies pioneering studies on the endocrine system and hormonal action. It began with observations linking the ovarian corpus luteum (CL) to pregnancy. The CL was initially described by Gabriele Falloppio in the mid-1500s, then Regnier de Graaf famously described the ovarian follicle in 1672. Meanwhile, the functional link between the CL and pregnancy was explored 200 years later by Emil Knauer. Gustave Jacob Born, an embryologist who for his part was interested in describing implantation, proposed that a substance produced by the CL “acts via the blood to protect the early embryo and facilitate its implantation in the uterus”. After that, multiple experiments were conducted by different researchers to demonstrate the relationship between the CL and pregnancy [7].

George W. Corner and Willard M. Allen initiated interest in the isolation and characterization of the hormone produced by the CL, noting that extracts of porcine CL had an effect on the endometrium in ovariectomized rats. Thus, in 1929, they managed to isolate an extract of porcine CL with the possibility of endometrial thickening from the alcohol partition of a compound with the chemical formula C_21_H_30_O_2_ (C = carbon, H = hydrogen, and O = oxygen). They named this component “progestin” based on its progestational activity [8,9].

In the United States in 1934, these authors produced crystalline progestin [10,11]. However, at the same time in Europe, three other groups [12,13,14] managed to produce the same compound. Of these groups, the participation of Adolf Butenandt stands out, who determined the delta-4-3-ketosteroid structure and was awarded the Nobel Prize in Chemistry in 1939 for his work on the structure of sex steroid hormones [12]. During the Second International Conference on the Standardization of Sex Hormones in 1935, Butenandt proposed that the CL extract be called “luteosterone” because it was produced in the CL; however, Allen remained adamant about the term “progestin”, and it was eventually named P4 [15]. Later, Butenandt continued his research, managing to synthesize P4 from pregnenolone and plant sterols. Finally, the pharmaceutical industry managed to produce compounds derived from P4 with progestational activity.

Between the 1930s and 1940s, the early and sustained administration of P4 was reported to lead to decreased gonadotropin secretion, inhibiting ovulation. In 1951, Gregory Pincus demonstrated that if a dose of >5 mg of P4 was administered to rabbits, it had a contraceptive effect, subsequently experimenting on female volunteers with unsatisfactory results. In 1943, Russell Marker discovered how to extract P4 from plant material. The process became known as “Marker degradation”, and it formed the basis for the production of synthetic hormones. In his eagerness to be able to produce more synthetic P4, he arrived in Mexico in 1951, where, by using the root of *dioscoreas pp*, he synthesized P4 from diosgenin [9,16]. He created the Syntex laboratory, where Luis Miramontes synthesized norethindrone in 1951. It was initially called 17α-ethinyl-19-nortestosteronase; however, at this stage, the molecule was not tested as a contraceptive. Subsequently, other 19-nor steroids were created, including norethynodrel, which Gregory Pincus, John Rock, and Celso-Ramón García demonstrated to have contraceptive efficacy in women who meticulously followed the indications [16].

**Figure 2:**
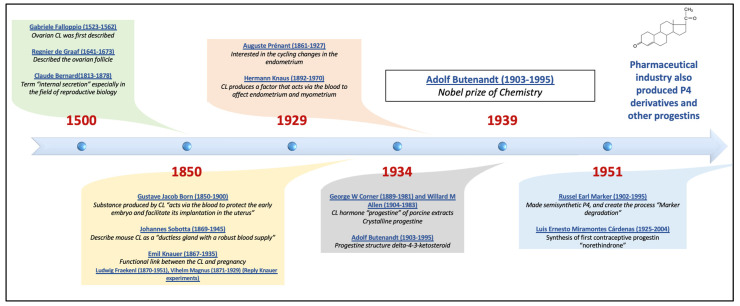
Timeline of progestin’s development, including the principal authors that contributed to the final isolation of progesterone, initially called progestin, which came from the discovery and study of the corpus luteum, and the discovery of progestin. P4 = progesterone, CL = corpus luteum.

Subsequently, research on the synthesis of various progestins with new properties continued, with their use initially being contraceptive but later encompassing the treatment of various disorders of reproductive endocrinology. Figure 2 shows the timeline of progestin’s development.

## 4. Mechanism of Action

Originally, progestogens were defined as compounds that maintain pregnancy; however, in humans, only P4 has this property. At present, the term progestogen is used to refer to all the compounds that promote the changes required for the establishment and maintenance of pregnancy, with P4 being the main natural progestogen. The term progestin refers to synthetic drugs [7]. The activity and potency of synthetic progestins are mostly evaluated via parameters associated with their endometrial effects [17].

In general, the actions of P4 and progestins result from their union with PRs. There are two PR isoforms (A and B), which have different distributions in tissues and orchestrate different actions. These isoforms arise from the different excision of a single gene that in humans is located on chromosome 11. They belong to the nuclear steroid receptor superfamily because their action is the result of genomic interactions [18,19]. The structure of the receptor is based on a DNA-binding domain near the amino-terminal, two highly conserved zinc fingers, a hinge region, a ligand-binding domain at the carboxy-terminal, and three functional domains (AF) that interact with coregulatory proteins near the amino-terminal and at the ligand-binding site. Once P4 interacts with the ligand-binding site and dimerizes into a progesterone response element (PRE) that interacts with the promoter of the gene, coregulators (coactivators or corepressors) are recruited into a transcriptional complex to activate or silence gene expression, manifesting a response in minutes to hours [20,21]. Some authors have provided evidence of the expression of a third isoform (PR C), which is overexpressed in myometrial cells, that does not have a DNA binding domain and does not have the capacity to bind to DNA but is able to bind to progesterone and may inhibit PRs, sequestering available progesterone away from the PR B isoform [22,23,24].

PR A and B have different functions and distributions, and when they are expressed in the same tissue, isoform A is dominant over isoform B. They interact with other receptors such as the estrogen receptor (ER), androgen receptor (AR), glucocorticoid receptor (GR), and mineralocorticoid receptor (MR), and they have the ability to involve different co-activators or co-repressors and thus have agonistic or antagonistic effects via these specific interactions with receptors [19,25,26,27].

The potency of progestins is basically classified by three specific actions, which are the main actions that allow progestins to be useful in different conditions. They include the following:

(1) Progestational activity: ability to transform the endometrium into the secretory phase and maintain a pregnancy.
(2) Anti-estrogenic activity: ability to downregulate the ER and consequently decrease the thickness of the estrogen-primed endometrium.
(3) Anti-androgenic activity: ability to block T from binding to AR, decreasing the androgen effect and antagonizing 5α-reductase [28].

The biological action of progestogens depends on the presence of estrogens (at least in most tissues) since their presence is key for PR expression; however, progestogens downregulate the expression of the RE. The decrease in ER expression and the activation of 17β-Hydroxysteroid dehydrogenase type 2 (an enzyme that catalyzes the conversion of estradiol to estrone) and estrone sulfotransferase (an enzyme that promotes conjugation of estrone) are effects induced by progestogens and progestin in the endometrium to which their anti-estrogenic effects are attributed [3].

Other effects of the progestins are related to their interactions with receptors, such as AR, GR or MR, inducing possible side-effects, such as acne, hyperlipidemia, salt and water retention, bloating, and usually as an antagonist of MR, decreased water retention and weight [28,29]. Some progestins have an anti-androgenic effect by competitive inhibition of the AR or binding to the enzyme 5-α reductase and hence interact with the conversion of testosterone (T) into dihydrotestosterone. Furthermore, when combined with estrogen, the non-androgenic progestins do not prevent the estrogen-dependent increase in the binding of sexual hormones to globulin, which later results in more binding of the circulating androgens and less free T being available [28].

**Figure 3:**
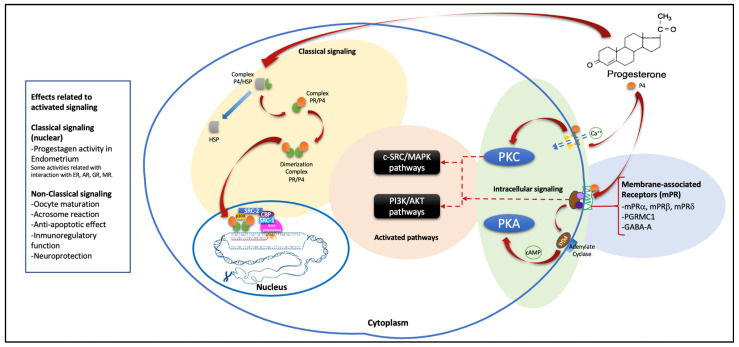
General scheme that summarizes the main described signaling pathways of P4. The classic pathway is shown with a yellow background, which is used by the PR A and PR B receptor isoforms. It is bound to HSP in the cytoplasm and, interacting with its ligand releases this protein, generating the PR/P4 complex, which dimerizes to create PR E. This subsequently binds with DNA and initiates the recruitment of coactivator or corepressor proteins to promote transcription and, subsequently, protein synthesis. On the blue, green, and orange backgrounds, the described non-classical pathway is shown in a very general and summarized way, which on the blue background encompasses the membrane receptors that are associated with protein G. The green background shows the pathways of intracellular signaling that are included in the PKC, which favors the flow of intracellular calcium, and the PKA, which favors the production of cAMP by the action of the receptor coupled to the G protein; this situation favors the signaling pathways shown with the orange background, which also involve nuclear interactions that favor effects related to these non-classical pathways. P4 = progesterone, PR = progesterone receptor, HSP = heat shock protein, PGRMC1 = progesterone receptor membrane component 1, GABA-A = gamma amino butyric acid receptor, PGα = G alpha protein, cAMP = cyclic adenine monophosphate, Ca^++^ = calcium, PKC = protein kinase C, PKA = protein kinase A, PI3K/AKT = phosphatidylinositol-3 kinase/protein kinase B, c-SRC/MAPK = non-receptor tyrosin kinase/mitogen activated protein kinase, SRC = steroid receptor coactivator, CBP = cAMP response element binding (CREB) binding protein, ER = estrogen receptor, AR = androgen receptor, GR = glucocorticoid receptor, MR = mineralocorticoid receptor.

In the same way, non-genomic effects related to P4 have been described, which differ from their genomic actions since they are very fast effects. They are found in cells at specific sites and are not inhibited by nuclear steroid receptor inhibitors since their activity is due to their binding to PR membranes, which belong to the family of progestin and adipoQ receptors (also called PAQRs) [1,30]. This union has been linked with different physiological actions such as acrosomal reaction, oocyte maturation, immunoregulatory function, antiapoptotic effects, steroidogenesis, neuroprotection, etc. These effects are achieved after favoring various signaling pathways related to the activation or inhibition of G proteins, the intracellular flow of calcium, and increases or decreases in the production of cyclic adenine monophosphate (cAMP) [30]. Another type of receptor, currently known as P4 receptor membrane component 1 (PGRMC1), has been described mainly in terms of its role in ovarian carcinogenesis due to its antiapoptotic effect and its potential association with tumor invasion and metastasis [30,31]. However, this review focuses on its genomic effects and its interaction with other nuclear receptors due to its usefulness in the treatment of various diseases in the field of reproductive endocrinology, and thus we do not focus on the non-genomic effects of P4 (which are very extensive and deserve a review with an individual approach to them). Figure 3 shows the classical and non-classical P4 mechanisms of action and their associated pathways.

## 5. Classification of Progestins

Knowledge of the chemical structure of P4 and progestins is essential to understand how each of them has different pharmacokinetics and potencies. We are able to divide the effects into compounds related to P4 and progestins [32]. There is a requirement for these steroids to have a progestogenic effect, which is the existence of a 3-keto group and a double bond between carbon (C) 4 and C5 in the A ring of the chemical structure (Δ4-3-keto group). Progestins that do not have this basic structure are prodrugs that are eventually converted to other active compounds from the same group (e.g., desogestrel or norgestimate) after oral administration [33,34].

A very useful way to classify progestins is based on their chemical structure, which can be carried out based on the presence or absence of common chemical groups. The basic structures of most progestins are structurally linked to either P4 or T and are broadly classified as pregnanes, norpregnanes, estranges, and gonanes, based primarily on their primary structures. The progestins that are structurally related to P4 can in turn be divided as follows, according to the presence of methyl groups in different positions:

○ Pregnanes: This group can be distinguished by a methyl group at position C10.
○ Norpregnanes: This group includes 19-norprogesterone derivatives and is characterized by the absence of a methyl group at C10 (or lack of C19).

This group or progestins related to P4 in turn is made up of compounds that may or may not have an acetate group.

In contrast, the progestins related to T can be further subdivided based on ethynyl groups into those that have an ethinyl group at C17 and those that lack this group:

○ Estranes: This group is called ethinylated progestins, and they lack an ethyl group at C13.
○ Gonanes: These are the so-called 13-ethylgonanos, which have an ethyl group at C13 of the basic steroid nucleus.
○ Non-ethylinated: These do not have an ethyl group.

**Figure 4:**
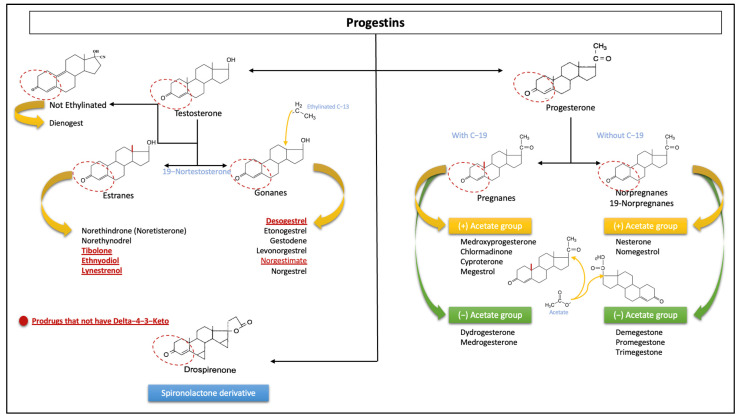
Classification of progestins according to their chemical structures. The sections with the dotted lines show the prerequisite group Δ4-3-Keto to bind the progesterone receptor and the general differences in C19 and C13 according to the different progestins’ origins. The red and underlined words refer to prodrugs that do not have the classical group Δ4-3-Keto. These include the unique derivative of spironolactone. Note: each molecule has independent characteristics with respect to others in the same group; the structure of each is not shown.

It is important to note that the classification scheme described does not imply the steroid precursor from which the progestin is derived (see Figure 4) [4,27,28,32,35].

**Figure 5:**
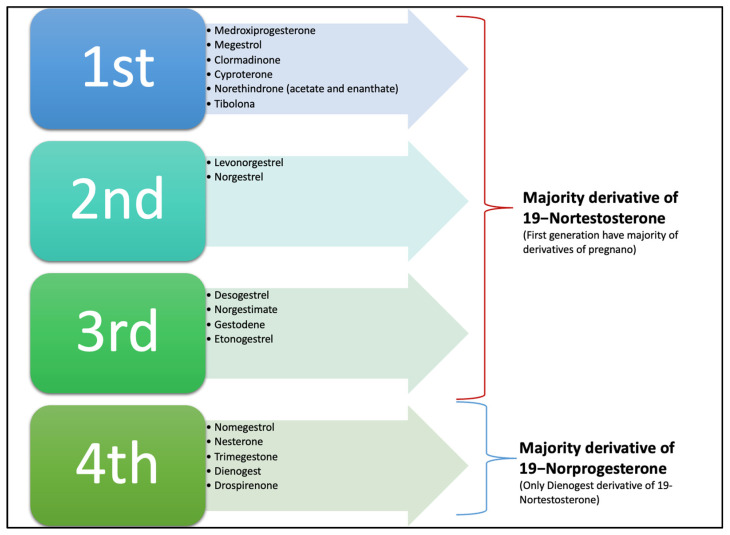
Classical classification of progestin according to generation; in the first three, the majority of compounds are derived from 19-Nortestosterone, and some are derivatives of pregnanes or 17-hydroxyprogesterone. The fourth generation are all derivatives of 19-Norprogesterone or Norgpregnane, except Dienogest, which is a derivative of ethylinated 19-Nortestosterone, and Gonane and Drospirenone, which are derivatives of Spironolactone. Note: Nowadays this classification is not widely used.

Additionally, progestins are conventionally classified into two groups, new and old progestins, which in turn are divided into three generations depending on their derivatives. The majority of progestins in all three generations are T-derived and therefore exhibit undesirable androgenic effects because, despite modifications, they still bind to the AR [36]. The newer, fourth-generation progestins have been developed to be closer in activity to P4 than progestins from the previous generations to avoid androgenic, estrogenic, mineralocorticoid, and glucocorticoid adverse effects (see Figure 5) [6,36].

The hormonal potency of steroids not only depends on their specific interactions with receptors but also with the local concentration of the free, active steroid. Many other endogenous and exogenous steroids or other factors may influence receptor-mediated activity. Therefore, the outcome of a comparative investigation into the potency of steroid hormones is largely dependent on the experimental conditions [34]. Moreover, binding to a distinct receptor might be associated with agonist, antagonist, or no hormonal activity (see Table 1) [33].

## 6. Clinical Use of Progestins

P4 and progestins with or without estrogen are often used to correct irregular bleeding and have also been used for the treatment of gynecological conditions, including dysfunctional uterine bleeding, oligomenorrhea, polymenorrhea, hypermenorrhea, dysmenorrhea, secondary amenorrhea, and endometriosis, as well as in menopausal hormone therapy, mainly due to its endometrial effects.

### 6.1. Effect in the Endometrium

P4 plays a very important role during the early luteal phase due to its effect on the endometrial transition from the proliferative to the secretory phase. The suppressive action of P4 over the ER of stromal and myometrial epithelial cells falls rapidly during the ovulatory phase. At the same time, due to the action of estradiol, PR expression increases exponentially in endometrial endothelial cells and then decreases dramatically in the late luteal phase [37]. In addition, progestogens in the endometrium decrease the number of ER, enhance the inactivation of estrogens, and, hence, inhibit estrogen-induced proliferation [17].

All these changes are the basis for the use of the combination of progestins in drugs with estrogen content in women with a uterus to promote the transformation of the endometrium and thus prevent hyperplasia [33] because estrogen treatments that are unopposed by progestin in women with a uterus increase the risk of endometrial hyperplasia dose- and time-dependently [38]. Therefore, the regular addition of progestin is mandatory to reduce this risk. However, the effect depends primarily on the treatment duration; at least 10–14 days per cycle is necessary to achieve sufficient protection [39,40].

The appropriate dose for either sequential or continuous combined therapy depends on the estrogen dose, the route of administration, the progestin potency, and the duration of the progestin addition. Moreover, it is also chosen while considering the cycle control or rate of irregular bleeding [41]. In continuous combined preparations, a lower dose of progestin might be sufficient for endometrial protection compared with sequential formulations [33].

### 6.2. Progestin in Endometriosis

Endometriosis is a chronic estrogen- and progestogen-responsive inflammatory condition defined by the presence of glands and stroma outside the uterine cavity. Its prevalence is approximately 7–10% of all women of reproductive age [42]. The clinical manifestations of endometriosis are pelvic pain and infertility. However, these symptoms are heterogeneous regarding both anatomic abnormalities and symptom severity [43].

The clinical observation of an apparent symptom resolution during pregnancy gave rise to the concept of treating patients with a pseudopregnancy regime. Initially, combinations of high dose estrogens and progestogens were used, but this was subsequently replaced by progestogens alone [42]. The medical treatment of endometriosis involves inducing decidualization and eventually atrophy or regression within a hormonally dependent ectopic endometrium. The effectiveness of progestins at treating endometriosis is not due its growth inhibiting actions alone but also to its induction of anovulation, inhibition of blood vessel growth, and anti-inflammatory actions [44].

Progestins also reduce the frequency and increase the amplitude of pulsatile gonadotropin-releasing hormone (GnRH), resulting in a reduction in follicle-stimulating hormone (FSH) and luteinizing hormone (LH) secretion. In this way, the continuous administration of progestins causes the suppression of ovarian steroidogenesis with anovulation and decreases sex hormone production in the ovaries [44,45]. Moreover, they can locally inhibit inflammatory pathways and responses and provoke the apoptosis of endometriotic cells. It has also been observed that interleukin-6, interleukin-8, and monocyte chemotactic protein type 1 secretions are reduced in stromal cells, and the cellular proliferation stimulated by tumor necrosis factor-α is decreased. Furthermore, they reduce oxidative stress, inhibit angiogenesis, and suppress the expression of matrix metalloproteinases, which may contribute to the development and growth of endometriotic lesions [45].

As was mentioned above, progestins are effective at resolving pain caused by endometriosis; the suggested mechanisms are listed below [46]:

- They reduce estrogen synthesis due to inactivation of the hypothalamic–pituitary–gonadal axis.
- They bind to the ER competitively, providing an anti-estrogenic effect.
- They inhibit the enzyme aromatase (CYP19A1).
- They activate 17-hydroxysteroid dehydrogenase type 2.
- They directly inhibit nuclear factor kappa β, which plays a key role in the processes of inflammation and neoangiogenesis.
- They cause the decidualization of stromal cells, and the secretory transformation of endometrial epithelial cells, which result in endometrial atrophy.

In a Cochrane Review, Brown et al. [42] did not find differences in the effectiveness at reducing all symptoms of endometriosis between dydrogesterone, medroxyprogesterone oral and depot, cyproterone acetate, desogestrel, or dienogest, but the authors conclude that the data should be interpreted with caution due to the limited number of trials and small sample sizes. Currently, only some progestogens are recommended for the treatment of endometriosis: medroxyprogesterone acetate (MPA), dienogest, dydrogesterone, and norethindrone acetate [42,43,46,47].

The current recommendations are to use combined contraceptives as the first choice, preferably with the lowest possible amount of ethinylestradiol (10–20 mcg), to choose cyclic combinations over continuous regimens [43] and to use progestins alone as a second line when the combination of estrogens and progestins is not effective or not tolerated. Progestins in intrauterine devices should be considered as a second or third line of treatment [44]. Treatment must be individualized depending on the formulations available in each country.

### 6.3. Progestin in Contraception

All contraceptive progestins possess a double action mechanism: central and peripheral.

- Central action: Inhibiting the secretion of gonadotropic hormones from the pituitary gland (especially LH) and, as a consequence, the inhibition of ovulation (depends on the dose of gestagen in the tablet) [46].

In synergy with ethinylestradiol, progestin reduces the release of gonadotropins in a time-dependent manner. This inhibitory action is mainly hypothalamic. Physiologically, P4 decreases the number of LH pulses. It is noteworthy that, at least with a low dose of an oral contraceptive, the suppression of gonadotropins is not observed immediately after the initiation of treatment, although a direct interaction with ovarian steroidogenesis might contribute to the modification of follicular maturation. It is important to notice that the inhibitory effect of progestins does not seem to depend of the amount of estrogen present in combined oral contraceptives [48].

- Peripheral action: Increasing the viscosity of the cervical mucus (by reducing the crypt volume, thickening the cervical mucus, and reducing the sialic acid content in the mucus and sperm activity) has specific effects on the endometrium (progestins suppress the mitotic activity of the endometrium, promoting its premature secretory transformation) and decreases the contractile activity of the fallopian tubes (by reducing the contractility and excitability threshold of the smooth muscle cells of their walls) [46].

In combined oral contraceptive pills, the estrogen doses are not sufficient to prevent ovulation. However, the progestin component or progestin alone suppresses GnRH, leading to a decrease in FSH and LH. Furthermore, it has been proven that even when administered at doses that do not constantly inhibit ovulation, progestin is still effective as a contraceptive acting in the cervical mucus and partially in the endometrium. The estrogen component potentiates progestin action and stabilizes the endometrium, reducing breakthrough bleeding [37]. Orally, progestins significantly decrease the amount, ferning, and spinnbarkeit of cervical mucus, lowering receptivity to sperm and sperm penetrations. Locally (in utero), progestins acts as a contraceptive without inhibiting ovulation, acting in the cervical mucus to inhibit sperm mobility and prevent endometrial proliferation.

In the endometrium, locally released progestogen eventually suppresses the proliferative activity via a diffuse pre-decidual reaction in the stroma, leading to poorly developed glands. The endometrium appears inactive, with a thin epithelium and a decidualized stroma. The presence of levonorgestrel causes a high expression of insulin-like growth factor binding protein-1 in the endometrium, thereby inhibiting the activity of insulin-like growth factor type 1 and suppressing endometrial proliferation. Vascular changes are also present and include the thickening of the arterial walls, the suppression of the spiral arteries, and capillary thrombosis [48].

Over time, various progestins have been developed in formulations for contraceptive purposes with minimal risk of side effects. The first generation of 19-nortestosterone derivatives had androgenic side effects. Later, different progestins with potent anti-androgenic effects became available. The most potent antiandrogenic progestins are cyproterone acetate, dienogest, drospirenone, and chlormadinone acetate. These molecules are antagonists of 5α-reductase, and they also block T from binding to AR, decreasing androgen’s effect. Drospirenone, as an analog of spironolactone, has anti-mineralocorticoid and anti-androgenic effects and has the potential to reduce blood pressure and low-density lipoprotein levels and to enhance high-density lipoprotein (HDL) levels [37].

Progestins alone have acquired relevance, especially in the initiation of contraception in the postpartum period, since women can restart their sexual life in the first weeks postpartum, and with the use of these contraceptives, an increased risk of stroke, myocardial infarction, or deep vein thrombosis has not been identified. In addition, their administration during lactation has not been described to affect the volume or composition of milk or to have any adverse effects on the infant. These contraceptives should be started within the first three weeks postpartum to maximize their effectiveness [49,50].

### 6.4. Progestin in Hormone Replacement Therapy

Typically, HRT includes an estrogen and progestogen combination mimicing ovary hormone production. Hormone replacement can be used in women with premature ovarian failure, menopause, and secondary (pituitary) or tertiary (hypothalamic) hypogonadism [37].

Progestins, along with estrogen, can be used in postmenopausal women with an intact uterus for HRT to relieve the symptoms associated with menopause (vasomotor symptoms or genitourinary symptoms) and to prevent osteoporosis. As was previously mentioned, the unopposed estrogen effect at the endometrial level can consequently generate endometrial hyperplasia or malignancy in a woman with an intact uterus, so these women should receive progestogen for endometrial protection [37,51]. Some points that must be considered in the use of progestins for endometrial protection in HRT are the duration and dose of the progestin, which in turn depend on the individual potency of each progestin; the duration of the progestogen sequence has been estimated to be at least 10–14 days per month, depending on the duration of the estrogens [39,40]. The dose of progestogen in HRT required to avoid the risk of hyperplasia relies on its potency, and it has been concluded by some authors that 19-norpregnanes derivatives have the greatest potency, followed by MPA, dydrogesterone, and micronized P4 (see Table 2) [52].

Initially, HRT was prescribed sequentially; however, due to breakthrough bleeding, continuous combined treatment was offered to women that did not wish to bleed. Several studies have shown that continuous combined treatment offers superior endometrial protection in comparison with sequential treatment [40].

The molecular differences between progestogens differentiate their clinical effects. Common side effects include fluid retention, bloating, breast tenderness, headaches, mood changes, and nausea [51]. Furthermore, all synthetic progestins used for HRT attenuate some or all of the beneficial estrogen-related increases in HDL as a result of androgen-mediated increases in hepatic lipase activity and increased degradation of HDL. In order to prevent HDL reduction, lowering the dose or the androgenicity of the progestin component of HRT has been proposed [54]. Additionally, progestogens with higher androgenic and glucocorticoid activity may interfere with the lipid profile and glucose tolerance. If the use of P4 is required, micronized P4 is considered the safer option. When compared with MPA, it has better outcomes in cardiovascular effects, blood pressure, venous thromboembolism, stroke, and breast cancer [37,52].

A proper clinical evaluation, choosing an adequate dose, and the estrogen/progestin combination are of pivotal importance to maximize the beneficial effect of HRT, especially if given within a reasonable time after menopause to women who need the therapy for the relief of menopausal symptoms [53]. Table 1 and Table 2 summarize the principal progestins and progestogens and their predominant effects and affinities.

### 6.5. Progestin in Assisted Reproductive Techniques

The endometrium is receptive to implantation for only a few days of the cycle before decidualization, between days 6 and 10 after the LH surge and ovulation [55]. P4 reduces the ER alfa levels and increases the expression of PR, thereby increasing endometrial sensitivity to P4 and endometrial receptivity. Progestins also increase the endometrium’s thickness, which is an important factor for ensuring successful embryo implantation [46].

In assisted reproductive techniques (ART), due to the drugs used in most hormonal cycles, there is a failure of the luteal phase, so it is logical that drugs with progestational activity or direct P4 are used in women with infertility during ART when superovulation is stimulated or in cryopreserved embryo transfer programs [46]. Some of the examples of ART procedures that iatrogenically cause a low P4 environment are in cycles where GnRH analogues are used because the reduction in gonadotropins will cause inadequate CL function, and another example is during follicle aspiration, where a substantial number of granulosa cells around the oocyte can be removed, leading to luteal insufficiency. Additionally, supraphysiological estradiol and P4 in the early luteal phase may trigger negative feedback, reducing LH levels and generating a dysfunctional CL. Therefore, progestogen supplementation during the luteal phase is a logical step to improve the chances of success [55,56].

Additionally, miscarriage and infertility are closely related conditions, and there is an overlap in the effect of progestogens on both. Vaginal or intramuscular P4 or oral dydrogesterone supports the luteal phase [56]. A Cochrane review found no differences in the clinical pregnancy rate in eight studies that compared intramuscular with micronized vaginal P4 [57]. Furthermore, in LOTUS 1 [58] and LOTUS 2 [59], which compared dydrogesterone to micronized vaginal P4 tablets and gel, no differences were found between the two. There are no studies comparing dydrogesterone to intramuscular P4 [56].

The proper time to start progestogen supplementation is between the evening of ovum retrieval and day three after oocyte retrieval. A premature elevation in P4 (>1.5 ng/mL) has been shown to lead to the dysregulation of gene expression and proteins involved in endometrial receptivity and implantation, and the cessation of therapy depends on the desired endpoint of treatment, the clinical pregnancy rate, or the live birth date [56]. The current recommendation is that luteal phase support should be stopped when a positive β fraction of the human chorionic gonadotropin test is achieved [60,61]. However, in patients with previous or recurrent miscarriages, it may be necessary to prolong the administration of progestogen for up to 12 weeks [62,63].

## 7. Summary and Conclusions

P4 is produced by several glands, but in women in particular, it is produced in the CL, and it is the dominant hormone after ovulation in the luteal phase. Rising P4 levels in the early luteal phase play an important role in the transition of the endometrium from the proliferative to the secretory phase. These changes are necessary for normal endometrial development, reproductive functioning, and pregnancy maintenance.

Progestogen refers to any synthetic P4 (progestin) or natural P4 with progestational effects. The preference for its use depends on the desired effect, whether as a contraceptive, for HRT, or for the treatment of endometriosis.

The proper choice of progestins must be individualized according to the desired effects, as well as a patient’s current diseases and cardiovascular risk. There is a lack of research in this field, and more is required to remove the stigma that hormonal compounds have gained, especially after the publication of the WHI, since there are currently different compounds with very different properties from those used in the classic studies that could offer benefits for different diseases and improve patients’ quality of life.

In this review, we mention some of the conditions in which these drugs are used, focusing mainly on conditions where their endometrial effects are beneficial. However, other properties of progestogens, especially those related to memory and metabolism, should be considered when making decisions regarding integrated management.

## Figures and Tables

**Table 1:** Spectrum of hormonal activities of progestogens.

Progestogen	AE	Est	And	AA	Glu	AM
Progesterone	++	-	-	(+)	+	+
Chlormadinone acetate	+	-	-	+	+	-
Cyproterone acetate	+	-	-	+	+	-
Medroxyprogesterone	+	-	(+)	-	+	-
Medrogestona	+	-	-	-	?	-
Dydrogesterone	+	-	-	-	?	(+)
Norethisterone	+	+	+	-	-	-
Levonorgestrel	+	-	+	-	-	-
Gestodene	+	-	+	-	(+)	+
Etonogestrel	+	-	+	-	(+)	-
Norgestimate	+	-	+	-	?	?
Dienogest	+	-	-	+	-	-
Tibolone	+	+	++	-	-	-
Drospirenone	+	-	-	+	?	+
Trimegestone	+	-	-	(+)	-	(+)
Promegestone	+	-	-	-	+	-
Nomegestrol	+	-	-	+	-	-

Abbreviations: AE = antiestrogenic, Est = estrogenic, Additionally, = androgenic, AA = antiandrogenic, Glu = glucocorticoid, AM = antimineralocorticoid. ++ = strongly effective, + = effective, (+) = weakly effective, - = ineffective, ? = unknown. Adapted from Wiegratz et al. [33].

**Table 2:** Progestin properties with their main group.

Progestational Activity	19-Norpregnanes *
Antiestrogenic	19-norpregnanes
Androgenic	Gonanes
Antiandrogenic **	Cyproterone acetate > Dienogest Drospirenone > Trimegestone
Antimineralocorticoid	Drospirenone
Glucocorticoid	Medroxyprogesterone acetate

* Without acetate group > with acetate group. ** Ordered by potency for this effect. Adapted from Nath et al. [53].

## Data Availability

Not applicable.
